# Improving young adult knowledge and attitudes towards reducing dementia risk

**DOI:** 10.1002/alz.085170

**Published:** 2025-01-09

**Authors:** Sharon A Savage, Eloise Trotter

**Affiliations:** ^1^ University of Newcastle, Callaghan, NSW Australia

## Abstract

**Background:**

Dementia may not be imminent for most young adults, however, lifestyle choices in early and midlife can play a significant role in dementia risk. Improving knowledge around modifiable factors and encouraging protective behaviours across the lifespan provides one important approach to lowering dementia incidence. This study aimed to test whether a short educational video, developed specifically for young adults, could improve knowledge of dementia risk factors and shift perceptions around when behaviour change may be relevant.

**Method:**

An online, randomised controlled study was conducted with 88 Australian, aged 18‐24 years. Participants were allocated to view one of two 5‐minute, age‐targeted health videos: “Let’s Talk Dementia” (n = 41) or “Let’s Talk Health” (n = 47). Videos discussed health facts and provided easy‐to‐implement actions young adults could take to protect their future health. Participants were assessed prior to and immediately after watching the video. Assessment involved answering true/false statements regarding modifiable dementia risk factors and indicating the age at which participants believed it was important to make lifestyle changes to reduce dementia risk.

**Result:**

At baseline, no differences were present between the two groups. Knowledge of dementia risk factors was limited (overall mean accuracy of 42%). Only 27% of participants showed awareness regarding the risk of hearing loss in midlife. Approximately 40% were unaware of the risks of hypertension and hyperlipidaemia. Following the video presentations, both groups improved their knowledge, although the greatest change was observed in the dementia video group (up to 76% accuracy vs 58% accuracy for general health video, t(87) = 4.24, p <.001). Here, more than 90% of participants now showed awareness of the risks of smoking, hypertension, and hyperlipidaemia, with 63% responding correctly regarding hearing loss. Majority of participants (69%) already indicated positive attitudes towards implementation of risk‐reducing behaviours by age 30, but increased to 93% of participants at immediate post‐intervention.

**Conclusion:**

Age‐targeted educational videos appear a promising method to increase knowledge of dementia risk factors and encourage positive attitudes towards healthy lifestyle choices in young adults. Further investigation is now needed to understand motivational and behaviour change mechanisms to ensure knowledge is put into action.

